# *tert*-Butyl­aminium phosphite

**DOI:** 10.1107/S1600536809006266

**Published:** 2009-02-28

**Authors:** Fang-Fang Jian, Jun-Li Zhang

**Affiliations:** a New Materials and Function Coordination Chemistry Laboratory, Qingdao University of Science and Technology, Qingdao 266042, People’s Republic of China

## Abstract

In the title compound, C_4_H_12_N^+^·H_2_PO_3_^−^, the components are linked by inter­molecular N—H⋯O and O—H⋯O hydrogen bonds, resulting in a two-dimensional framework.

## Related literature

For general background, see: Rao *et al.* (2000 ▶); Wang *et al.* (2002 ▶). For related structures, see: Loub *et al.* (1978 ▶); Smolin *et al.* (2003 ▶).
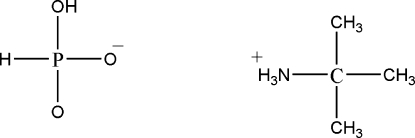


## Experimental

### 

#### Crystal data

C_4_H_12_N^+^·H_2_PO_3_^−^
*M* *_r_* = 155.13
Monoclinic, 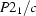

*a* = 7.621 (2) Å
*b* = 6.561 (2) Å
*c* = 17.545 (5) Å
β = 111.10 (3)°
*V* = 818.5 (4) Å^3^
*Z* = 4
Mo *K*α radiation
μ = 0.28 mm^−1^
*T* = 295 K
0.2 × 0.15 × 0.11 mm

#### Data collection

Enraf–Nonius CAD-4 diffractometer
Absorption correction: none
4275 measured reflections
1524 independent reflections
1332 reflections with *I* > 2σ(*I*)
*R*_int_ = 0.023
3 standard reflections every 100 reflections intensity decay: none

#### Refinement

*R*[*F* ^2^ > 2σ(*F* ^2^)] = 0.030
*wR*(*F* ^2^) = 0.084
*S* = 1.06
1524 reflections
103 parameters
H atoms treated by a mixture of independent and constrained refinement
Δρ_max_ = 0.19 e Å^−3^
Δρ_min_ = −0.24 e Å^−3^

### 

Data collection: *CAD-4 Software* (Enraf–Nonius, 1989 ▶); cell refinement: *CAD-4 Software*; data reduction: *NRCVAX* (Gabe *et al.*, 1989 ▶); program(s) used to solve structure: *SHELXS97* (Sheldrick, 2008 ▶); program(s) used to refine structure: *SHELXL97* (Sheldrick, 2008 ▶); molecular graphics: *SHELXTL/PC* (Sheldrick, 2008 ▶); software used to prepare material for publication: *WinGX* (Farrugia, 1999 ▶).

## Supplementary Material

Crystal structure: contains datablocks global, I. DOI: 10.1107/S1600536809006266/hk2620sup1.cif

Structure factors: contains datablocks I. DOI: 10.1107/S1600536809006266/hk2620Isup2.hkl

Additional supplementary materials:  crystallographic information; 3D view; checkCIF report

## Figures and Tables

**Table 1:** Hydrogen-bond geometry (Å, °)

*D*—H⋯*A*	*D*—H	H⋯*A*	*D*⋯*A*	*D*—H⋯*A*
N1—H3*N*⋯O1^i^	0.90 (2)	1.93 (2)	2.826 (2)	173.1 (18)
N1—H2*N*⋯O3^ii^	0.94 (2)	1.91 (2)	2.8425 (19)	174.7 (17)
N1—H1*N*⋯O1	0.87 (2)	1.94 (2)	2.806 (2)	173.1 (19)
O2—H1*O*⋯O3^iii^	0.829 (10)	1.808 (10)	2.6339 (17)	174 (2)

Symmetry codes: (i) 
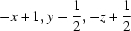
; (ii) 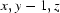; (iii) 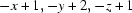.

**Fig. 1.**
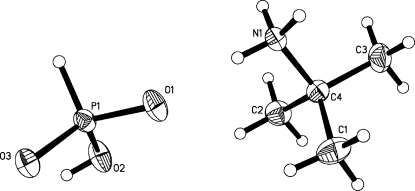
The molecular structure of the title molecule with the atom-numbering scheme. Displacement ellipsoids are drawn at the 30% probability level.

**Fig. 2.**
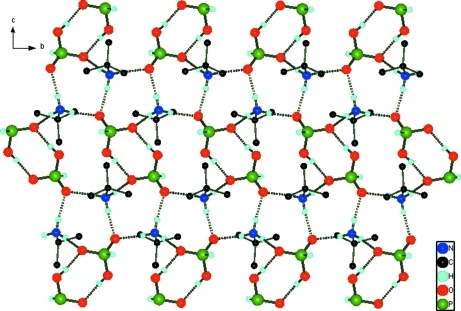
A stereoview of the crystal structure of the title compound. Showing the formation of channels and two-dimensional framework. For the sake of clarity, H atoms bonded to C atoms have been omitted. Hydrogen bonds are shown as dashed lines. Color scheme: C = black, O = red, N = blue, P = green, H = cyan.

## supplementary crystallographic information

### Comment

Recently, compounds containing the phosphorous acid group have attracted much interest because they exhibit some biological activities and the functions of intermediates in the formation of open-framework metal phosphates templated by organic amines (Rao *et al.*, 2000; Wang *et al.*, 2002). Though the structure of H_2_PO_3_ ion was described previously (Loub *et al.*, 1978), the ammonium phosphite zwitterion was only reported by Smolin (Smolin *et al.*, 2003). In order to search for new phosphite compounds with higher bioactivity, we synthesized the title compound and report herein its crystal structure.

In the title compound, (Fig. 1), the bond lengths and angles (Table 1) are generally within normal ranges (Smolin *et al.*, 2003). The NH_3_ group of alkyl is additionally protonated by an H atom of the phosphite ion to give a positively charged molecule. The phosphite ion is shaped like a tetrahedron. The H1P atom is localized at the P atom at a distance of 1.278 (19) Å, which is not involved in hydrogen bonding. The O2-P1 [1.5708 (15) Å] bond is significantly longer than the other P-O bonds of the tetrahedron (Table 1). Phosphite and amine molecules are linked by intramolecular N-H···O hydrogen bonds (Table 2).

In the crystal structure, intermolecular N-H···O and O-H···O hydrogen bonds (Table 2) link the molecules (Fig. 2). Each two orthophosphorous acids are linked by O-H···O hydrogen bonds into channels, while the orthophosphorous acids and amine molecules are linked by N-H···O hydrogen bonds into chains. Then, the chains are linked by N-H···O and O-H···O hydrogen bonds into a two-dimensional framework, as in the phosphates reported by Smolin (Smolin *et al.*, 2003), in which they may be effective in the stabilization of the structure.

### Experimental

The title compound was prepared by the reaction of phosphorous acid (0.164 g, 2.0 mmol) and *tert*-butylamine (0.182 g, 2.5 mmol) stirred in water/ethanol (5:1 *v*/*v*) solution (20 ml). Single crystals suitable for X-ray analysis were obtained by recrystallization from water/ethanol (5:1 *v*/*v*) solution at room temperature over a period of 3 d.

### Refinement

H1N, H2N, H3N (for NH_3_), H10 (for OH) and H1P (for PH) were located in difference synthesis and refined isotropically [N-H = 0.87 (2)-0.94 (2) Å, U_iso_(H) = 0.041 (5)-0.052 (6) Å^2^; O-H = 0.829 (10) Å, U_iso_(H) = 0.063 (7) Å^2^ and P-H = 1.278 (19) Å, U_iso_(H) = 0.046 (5) Å^2^]. The remaining H atoms were positioned geometrically with C-H = 0.96 Å, for methyl H atoms and constrained to ride on their parent atoms, with U_iso_(H) = 1.5U_eq_(C).

### Crystal data

**Table:**

C_4_H_12_N^+^·H_2_PO_3_^−^	*F*(000) = 336
*M**_r_* = 155.13	*D*_x_ = 1.259 Mg m^−^^3^
Monoclinic, *P*2_1_/*c*	Melting point: 504.8 K
Hall symbol: -P 2ybc	Mo *K*α radiation, λ = 0.71073 Å
*a* = 7.621 (2) Å	Cell parameters from 25 reflections
*b* = 6.561 (2) Å	θ = 4–14°
*c* = 17.545 (5) Å	µ = 0.28 mm^−^^1^
β = 111.10 (3)°	*T* = 295 K
*V* = 818.5 (4) Å^3^	Block, colorless
*Z* = 4	0.2 × 0.15 × 0.11 mm

### Data collection

**Table:**

Enraf–Nonius CAD-4 diffractometer	*R*_int_ = 0.023
Radiation source: fine-focus sealed tube	θ_max_ = 25.5°, θ_min_ = 2.5°
graphite	*h* = −9→7
ω scans	*k* = −7→7
4275 measured reflections	*l* = −21→19
1524 independent reflections	3 standard reflections every 100 reflections
1332 reflections with *I* > 2σ(*I*)	intensity decay: none

### Refinement

**Table:**

Refinement on *F*^2^	Secondary atom site location: difference Fourier map
Least-squares matrix: full	Hydrogen site location: inferred from neighbouring sites
*R*[*F*^2^ > 2σ(*F*^2^)] = 0.030	H atoms treated by a mixture of independent and constrained refinement
*wR*(*F*^2^) = 0.084	*w* = 1/[σ^2^(*F*_o_^2^) + (0.0424*P*)^2^ + 0.222*P*] where *P* = (*F*_o_^2^ + 2*F*_c_^2^)/3
*S* = 1.06	(Δ/σ)_max_ < 0.001
1524 reflections	Δρ_max_ = 0.19 e Å^−^^3^
103 parameters	Δρ_min_ = −0.24 e Å^−^^3^
0 restraints	Extinction correction: *SHELXL97* (Sheldrick, 2008), Fc^*^=kFc[1+0.001xFc^2^λ^3^/sin(2θ)]^-1/4^
Primary atom site location: structure-invariant direct methods	Extinction coefficient: 0.040 (4)

### Special details

**Table:**

Geometry. All e.s.d.'s (except the e.s.d. in the dihedral angle between two l.s. planes) are estimated using the full covariance matrix. The cell e.s.d.'s are taken into account individually in the estimation of e.s.d.'s in distances, angles and torsion angles; correlations between e.s.d.'s in cell parameters are only used when they are defined by crystal symmetry. An approximate (isotropic) treatment of cell e.s.d.'s is used for estimating e.s.d.'s involving l.s. planes.
Refinement. Refinement of *F*^2^ against ALL reflections. The weighted *R*-factor *wR* and goodness of fit *S* are based on *F*^2^, conventional *R*-factors *R* are based on *F*, with *F* set to zero for negative *F*^2^. The threshold expression of *F*^2^ > σ(*F*^2^) is used only for calculating *R*-factors(gt) *etc*. and is not relevant to the choice of reflections for refinement. *R*-factors based on *F*^2^ are statistically about twice as large as those based on *F*, and *R*- factors based on ALL data will be even larger.

### Fractional atomic coordinates and isotropic or equivalent isotropic displacement parameters (Å^2^)

**Table:**

	*x*	*y*	*z*	*U*_iso_*/*U*_eq_	
P1	0.45578 (6)	0.77903 (6)	0.40886 (2)	0.03180 (18)	
H1P	0.308 (3)	0.691 (3)	0.4103 (11)	0.046 (5)*	
O1	0.51036 (19)	0.66932 (19)	0.34600 (7)	0.0445 (4)	
O2	0.6124 (2)	0.7419 (2)	0.49496 (8)	0.0496 (4)	
H1O	0.606 (3)	0.829 (3)	0.5280 (11)	0.063 (7)*	
O3	0.41488 (17)	1.00287 (17)	0.39392 (7)	0.0386 (3)	
N1	0.6062 (2)	0.2679 (2)	0.32177 (9)	0.0334 (3)	
H1N	0.574 (3)	0.389 (3)	0.3327 (12)	0.048 (6)*	
H2N	0.544 (3)	0.174 (3)	0.3436 (11)	0.041 (5)*	
H3N	0.563 (3)	0.246 (3)	0.2674 (14)	0.052 (6)*	
C1	0.9076 (3)	0.4138 (4)	0.32887 (14)	0.0620 (6)	
H1A	1.0419	0.4067	0.3547	0.093*	
H1B	0.8640	0.5427	0.3410	0.093*	
H1C	0.8732	0.4002	0.2708	0.093*	
C2	0.8688 (3)	0.2586 (3)	0.45276 (11)	0.0497 (5)	
H2A	1.0015	0.2371	0.4796	0.074*	
H2B	0.8010	0.1568	0.4702	0.074*	
H2C	0.8361	0.3914	0.4665	0.074*	
C3	0.8674 (3)	0.0346 (3)	0.33646 (13)	0.0538 (5)	
H3A	1.0000	0.0113	0.3628	0.081*	
H3B	0.7991	−0.0684	0.3531	0.081*	
H3C	0.8344	0.0292	0.2783	0.081*	
C4	0.8178 (2)	0.2434 (2)	0.36060 (11)	0.0372 (4)	

### Atomic displacement parameters (Å^2^)

**Table:**

	*U*^11^	*U*^22^	*U*^33^	*U*^12^	*U*^13^	*U*^23^
P1	0.0408 (3)	0.0290 (3)	0.0292 (3)	−0.00216 (17)	0.0170 (2)	−0.00246 (16)
O1	0.0689 (9)	0.0358 (7)	0.0335 (7)	0.0069 (6)	0.0242 (6)	−0.0032 (5)
O2	0.0685 (9)	0.0427 (8)	0.0329 (7)	0.0185 (6)	0.0124 (7)	−0.0020 (5)
O3	0.0508 (7)	0.0330 (7)	0.0332 (6)	0.0054 (5)	0.0168 (5)	0.0001 (5)
N1	0.0418 (8)	0.0286 (8)	0.0317 (8)	0.0013 (6)	0.0157 (7)	−0.0006 (6)
C1	0.0575 (13)	0.0658 (14)	0.0668 (14)	−0.0160 (11)	0.0272 (11)	0.0103 (11)
C2	0.0500 (11)	0.0570 (12)	0.0375 (10)	−0.0032 (9)	0.0103 (9)	−0.0018 (8)
C3	0.0512 (11)	0.0512 (12)	0.0612 (12)	0.0124 (9)	0.0229 (10)	−0.0050 (9)
C4	0.0369 (9)	0.0376 (9)	0.0385 (9)	−0.0009 (7)	0.0153 (8)	−0.0001 (7)

### Geometric parameters (Å, °)

**Table:**

P1—H1P	1.278 (19)	C1—H1B	0.9600
O1—P1	1.4958 (12)	C1—H1C	0.9599
O2—P1	1.5708 (15)	C2—C4	1.524 (3)
O2—H1O	0.829 (10)	C2—H2A	0.9600
O3—P1	1.5043 (12)	C2—H2B	0.9600
N1—C4	1.516 (2)	C2—H2C	0.9600
N1—H1N	0.87 (2)	C3—C4	1.521 (2)
N1—H2N	0.94 (2)	C3—H3A	0.9600
N1—H3N	0.90 (2)	C3—H3B	0.9600
C1—C4	1.517 (3)	C3—H3C	0.9600
C1—H1A	0.9600		
			
O1—P1—O2	108.54 (8)	C4—C2—H2A	109.4
O1—P1—O3	115.87 (7)	C4—C2—H2B	109.4
O1—P1—H1P	106.1 (8)	C4—C2—H2C	109.6
O2—P1—H1P	106.3 (8)	H2B—C2—H2A	109.5
O3—P1—O2	110.90 (7)	H2C—C2—H2A	109.5
O3—P1—H1P	108.5 (8)	H2C—C2—H2B	109.5
P1—O2—H1O	110.5 (16)	C4—C3—H3A	109.5
C4—N1—H1N	109.6 (13)	C4—C3—H3B	109.5
C4—N1—H2N	111.0 (11)	C4—C3—H3C	109.5
C4—N1—H3N	112.5 (14)	H3B—C3—H3A	109.5
H2N—N1—H1N	106.5 (16)	H3C—C3—H3B	109.5
H3N—N1—H1N	110.8 (17)	H3C—C3—H3A	109.5
H3N—N1—H2N	106.2 (16)	N1—C4—C1	107.70 (15)
C4—C1—H1A	109.6	N1—C4—C2	107.08 (15)
C4—C1—H1B	109.2	N1—C4—C3	107.52 (14)
C4—C1—H1C	109.5	C1—C4—C2	111.37 (16)
H1B—C1—H1A	109.5	C1—C4—C3	111.79 (17)
H1C—C1—H1A	109.5	C3—C4—C2	111.14 (15)
H1C—C1—H1B	109.5		

### Hydrogen-bond geometry (Å, °)

**Table:**

*D*—H···*A*	*D*—H	H···*A*	*D*···*A*	*D*—H···*A*
N1—H3N···O1^i^	0.90 (2)	1.93 (2)	2.826 (2)	173.1 (18)
N1—H2N···O3^ii^	0.94 (2)	1.91 (2)	2.8425 (19)	174.7 (17)
N1—H1N···O1	0.87 (2)	1.94 (2)	2.806 (2)	173.1 (19)
O2—H1O···O3^iii^	0.83 (1)	1.81 (1)	2.6339 (17)	174 (2)

Symmetry codes: (i) −*x*+1, *y*−1/2, −*z*+1/2; (ii) *x*, *y*−1, *z*; (iii) −*x*+1, −*y*+2, −*z*+1.
